# Croatian National Centre for Biobanking – a new perspective in biobanks governance?

**DOI:** 10.3325/cmj.2014.55.416

**Published:** 2014-08

**Authors:** Ana Borovečki, Luciana Caenazzo, Davor Ježek, Monika Karija-Vlahović, Branka Golubić

**Affiliations:** 1 University of Zagreb, School of Medicine, “Andrija Štampar” School of Public Health, Zagreb, Croatia; 2 Department of Molecular Medicine, University of Padua, Padua, Italy; 3 Department of Histology and Embryology University of Zagreb, School of Medicine, Zagreb, Croatia; 4 Department of Forensic Medicine and Criminology, DNA Laboratory, University of Zagreb, School of Medicine, Zagreb, Croatia; 5 University Hospital Centre Zagreb, Department of Transfusion Medicine and Cellular Therapy, Zagreb, Croatia

## Biobanks: European situation

Ethical issues in biobanking, as well as organization and management of biobanks, have become permanent topic of scientific publications in the last 15 years (1). The Expert Group of the European Commission Defines biobanks as collections of various types of biological samples (cells, tissues, blood, DNA) plus related databases. They can be small collections or large national repositories, population-based or disease specific, established for diagnostic, therapeutic (eg, transplantation), forensic, or research purposes (1-3).

Different countries in Europe have different types of biobanks, and the material is collected in different ways for different purposes (2-7) (Table 1). For example, there are different approaches to obtain informed consent depending on the type and the use of the collected tissue samples and whether the material is irreversibly anonymized or not. In biobanks with irreversibly anonymized samples, no information regarding the donor can ever be obtained from the sample. In these cases, no informed consent is obtained because the donor’s consent is presumed if he or she does not opt out. The same process is used in some European countries when leftover tissue is concerned (eg, Belgium, Denmark, and the Netherlands). The future donors receive the information about potential use of their leftover tissue and they are given the possibility to opt out.

**Table 1:** European biobanks

Type of bio bank	Purpose of the material collection	Type of tissue collected and storage	Countries
Population biobanks	population research	mainly blood (frozen samples)	Island, Estonia, Latvia, Hungary, Poland, United Kingdom
Biobanks oriented to specific diseases	research of specific disease	different types of tissue (frozen samples)	majority of European countries
Biobanks created during the course of clinical trails	research purposes	different types of tissue (frozen samples)	majority of European countries
Diagnostic archives	diagnosis of disease (mainly pathological archives)	different types of tissues (mainly paraffin blocks sometimes frozen samples)	all European countries
Forensic biobanks	forensic (criminalistics) purposes(identification of victims or crime perpetrator)	blood, urine, semen, bone tissues and other tissues (frozen samples)	majority of European countries
Transplantation biobanks (eye banks, cord blood banks)	therapeutic purposes (organ transplantation)	different types of tissue (frozen samples)	majority of European countries
Biobanks of leftover materials	research	different types of tissue (frozen samples)	some European countries eg, the Netherlands
Blood banks	therapeutic purposes (transfusion)	blood, frozen samples	all European countries

When it comes to the procurement of tissue for diagnostic purposes that may later be used for research or in sample collections exclusively created for research purposes, informed consent can be obtained in the several ways. In broad consent schemes, permission is given for the material to be used for all future research purposes. Partially restricted consent allows the use of the material in certain types of research studies done in a certain scientific filed. Specific consent allows the use of the obtained material only in one research project. Biobanks sometimes offer tiered or multi-layered consent. This means that each donor can choose whether the sample can be used in all types of research, only in certain studies, or only in one specific research project (2,7,8).

On the theoretical level, no consensus seems to exist which consent model fits best as the consent model for balancing the interests of donors and research. Recently a new approach for biobanking has been proposed – dynamic consent model. Dynamic consent uses modern communication strategies to inform, involve, offer choices, and obtain consent for every research project based on biobank resources. Such approaches usually use web-based platforms with an interface that allows interactive communication of research participants with custodians of biobanks (9,10).

The work of biobanks is monitored by experts based on quality standards in the field and by ethic of governance or ethics and governance committees. This new type of ethics committee reviews proposals for different projects connected with a certain biobank, protects the future research subjects, monitors and informs the public about the research and biobank functioning, approves date exchange and transfer, and revises guidelines and working frameworks regarding recruitment access or handling of complaints (5,11).

The legal basis for establishment of biobanks is left for each country to implement according to its own legal standards. Therefore the United Kingdom, Estonia, Sweden, Portugal, and Iceland have their own national legal standards that regulate the foundation and management of different biobanks (6,12). Other countries decided to draft national recommendations regarding biobanks (eg, Germany, Austria, Cyprus) (12,13). In addition, there are some common EU standards to be followed (13) (Table 2).

**Table 2:** Common European Union legal biobanking framework

**Lisbon Treaty** (title XIV devoted to public health Article (168) 4 promotes common safety standards of quality and safety when it comes to organs and substances of human origin, blood and blood derivatives)
**Directive 2004/23/EC, 2006/17/EC** (standards on quality, safety, donation, procurement, testing, processing, preservation, storage and distribution of human tissues and cells used in donation and serious adverse events that may be in connection to it)
**Directive 95/46/EC** (data protection)
**Additional Protocol to the Convention on Human Rights and Biomedicine concerning Biomedical Research**
**Additional Protocol to the Convention on Human Rights and Biomedicine concerning Genetic Testing for Heath Purposes**
**Opinion No 11 on Ethical aspects of human tissue biobanking from 1998 of the European Group on Ethics in Science and New Technologies**
**Council of Europe Recommendations: Recommendations No R(94)1 on human tissue banks**
**Recommendation Rec (2004) 4 of the Committee of Ministers to member states on research on biological materials of human origin,**
**Recommendation No R(92)1 on the use of DNA (DNA) within the framework of criminal justice system**

Since in 2011 an incentive for coordination of biobanks was proposed, several such projects have been launched (15), among which was Biobanking and Bimolecular Resources Research Infrastructure (BBMRI). The project recruited 270 associated organizations, mostly biobanks linking 20 million biological samples in 22 countries (14) (Table 3).

**Table 3:** European biobanking networks

Project name	Project aim
TUBAFROST	virtual tumor tissue bank of frozen tumor tissues
EROBIOBANK	network of biobanks connected with the research of rare disease (human DNA, cell and tissue samples) – 15 members from France, Germany, Hungary, Italy, Malta, Slovenia, and Spain
CONTICANET	connective tissue cancers biobanks network
BBMRI	pan European and internationally broadly accessible network of existing and de novo created biobanks and bimolecular resources
CNIO Tumour Bank Network	Spanish tumor hospital banks
Euro BoNeT project	primary bone tumor bank
Confederation of Cancer Biobanks	UK-based consortium for development, management and use of biobanking resources for cancer research
The NCI Office of Biorepositories and Biospecimen Research (OBBR)	established in 2005 for developing common bio-repository infrastructure

It is still not clear to what degree biobanks will be able to generate new knowledge that can be exploited and utilized in the health care sector and for development of products and services. Therefore, some biobanks will be forced to close or be substantially reconfigured since the benefits of biobanking are sometimes very difficult to be operationalized in economic terms. There are reports of biobanking managers who struggle with problems hoping their biobanks will exist permanently despite limited funding (16).

## Biobanks: Croatian situation

In Croatia there are several biobanking facilities intended for various purposes. In the 1980s Croatian Bone Marrow Donor Registry (CB-MDR) was established at the University Hospital Centre Zagreb, which performs sporadic HLA typing of family members in the cases of bone marrow transplantation. The Cord Blood Bank (CBB) was established in the same period at the University Hospital Center Zagreb. In 2007, Foundation “Ana Rukavina” was set up as a result of a large campaign following the death of a young female journalist. Foundation “Ana Rukavina” nowadays includes an allogeneic public cord blood bank and a registry of potential allogeneic bone marrow donors. At the University Hospital Center Zagreb, there is also a private cord blood bank for autologous use. Clear quality control standards, protocols for international exchange of samples and procurement, and informed consent procedures are in place based on international and national legal frameworks: EU Directives 2004/23/EZ, 2006/17/EZ, 2006/17/EZ, 2010/453/E, and the Law on Procurement and Transplantation of Human Body Parts for the Purpose of Therapeutic Procedures and its bylaws (17-19).

In Croatia, there is also a blood bank, which is a part of the Croatian Institute for Transfusion Medicine. The bank has regional organization structure and is based on general population’s voluntary donation of blood. The bank is well organized with high quality standards and legal provisions (laws and bylaws) in place: EU Directive 2005/61and Law on Blood and Blood derivates (20,21).

The first Croatian eye bank – Croatian Lions Eye Bank (LHOB) was founded at the Clinical Hospital “Sv. Duh” in Zagreb in the 1980s. LHOB acts in accordance with the standards of the European Association for Ocular Banking. It provides tissues for corneal transplantation and processes and stores amniotic membranes and sclera used in the surgical treatment of diseases of the anterior eye segment. Several years later, an eye bank was also founded at the University Hospital Center Zagreb. Their work is covered by the Law on Procurement and Transplantation of Human Body Parts for the Purpose of Therapeutic Procedures and its bylaws (22).

Diagnostic archives have been a permanent fixture of Croatian health care system as part of pathology departments in different health care institutions. They are well organized and regulated by legal provisions based on Health Protection Act (23).

Banks of frozen embryos and sperm have been present in Croatia for many years since the first introduction of artificial insemination and IVF procedures. They have been the object of debate and controversy for several years because of the lack of legal provisions regulating their work. Now their work is clearly ruled by the Law on Medically Assisted Procreation and its bylaws (24).

Croatia has been involved in DNA identification of the victims from the 1991-95 war in Croatia and the region since the beginning of the war. The result of these efforts are two forensic DNA laboratories at the Schools of Medicine at the University of Zagreb and Osijek, and one laboratory at the University Hospital Split. Their work is mandated by the Geneva Conventions and Rules on Collection of Biological Samples for DNA Analysis (25,26). All these collections form a bio-bank of DNA samples for identification purposes. The owner of the bio-bank is the Ministry of War Veterans. The contract with those laboratories is renewed every year by the Ministry, which procures their services and facilities in order to continue with this work and use the biobank (26).

Together with these forensic laboratories, the forensic laboratory at the Ministry of the Interior located at the Forensic Science Centre “Ivan Vučetić” and at least one private laboratory are capable of doing forensic DNA analysis. Their work in this area is regulated by the Criminal Procedure Act and Prison Sentence Act. In Croatia it is obligatory to take biological samples and perform forensic DNA analysis for criminal offenses with a minimum sentence of 6 months. Data collected through DNA analysis have to be stored for 20 years (25,27-29). Currently a new bylaw is in process of adoption concerning the procurement of biological samples and the modes of forensic DNA analysis, which aims to set additional quality standards and facilitate the establishment of a forensic DNA biobank. According to this proposal, all the forensic evidence can only be kept at the Forensic Science Centre of the Ministry of the Interior. The following questions remain: What will become of the samples and data already collected and stored by forensic laboratories at Universities? Will they be destroyed? Will they be transferred? Will other laboratories be able to also do DNA analysis or only the Forensic Centre of the Ministry of the Interior? Can private laboratories perform forensic DNA analysis?

Croatia also has a population biobank – The 100 001 Dalmatians Biobank. This is an isolated population biobank with a focus on genetic basis of complex diseases and a target sample of 100 001 individuals (30,31). Today it contains samples of about 4500 individuals from six Croatian islands (Vis, Korčula, Mljet, Lastovo, Rab, Susak) and the city of Split. Among other research biobanks at different research institutions and medical schools in Croatia, there is a mini-bank of testicular biopsies at the University of Zagreb School of Medicine (32,33). However, when it comes to population biobanks or different research biobanks there are no legal provisions regulating their work. Except for a private cord blood bank in Croatia there are also private DNA laboratories involved in DNA diagnostics and research with small DNA collections. A set of standards should be given regarding their organization, management, quality control, and conditions for international exchange of samples and procurement and informed consent (29).

## Croatian National Centre for Biobanking

Some biobanking facilities in Croatia are already well organized and some are still in the developing phase. The majority of the authors of this paper have been involved in the biobanking organization or research. Drawing on their experiences they came up with a way to improve the current Croatian biobanking situation by creating a national network of different Croatian biobanks – Croatian National Centre for Biobanking (Figure 1).

The Croatian National Centre for Biobanking would be a virtual network of all biobanks in Croatia coordinated by the representatives of different Croatian biobanks. The repositories would be left at the same places but the quality of their work would be monitored centrally. We believe that such approach would not require substantial financial resources because the already existing resources would be put under one roof preventing redundancy and improving the coordination between different biobanking facilities. The management structure would consist of a scientific advisory committee and an ethics governance committee, ie, the expertise already existing in different biobanks.

The Scientific Advisory Committee would consist of one member from each of the biobanks present in the consortium and four external experts coming from the field of biobanking in Croatia or outside of Croatia. Its main task would be to monitor the implementation and execution of quality control standards in procurement, storage, sample shipment, and scientific work done within the consortium of biobanks.

The Ethics Governance Committee would be in charge of implementing and monitoring the procedures of obtaining informed consent and dealing with other ethical challenges, such as the protection of vulnerable subjects, safeguarding of privacy, communication of research results to donors, conflicts over patenting, access, and the need for open science, the rights of donors to retain a property claim or control over their tissues, and the management of samples collected from vulnerable groups. Furthermore, one of the tasks of the Ethics Governance Committee would also be the provision of the transparent information about the work and research done within the biobanking consortium to the general public. Finally, it would be involved in a campaign of ethical education to ensure that all researchers involved in biobanks are aware of their ethical responsibilities, and that donors are better informed of ethical aspects of donation. The committee would consist of five independent experts coming from the fields of ethics, law, and biobanking and two members of general public.

The two committees would meet at least twice year at the premises of one of the consortium members to discuss the current work and problems within the consortium. They would annually submit their reports to the Ministry of Health, Ministry of Science, Education and Sport, Ministry of War Veterans, and the Ministry of the Interior, since the biobanks in the consortium are related to their respective areas of work.

Croatian National Centre for Biobanking requires improved legal provisions, especially for research biobanks, population biobanks, and private DNA laboratories in particular. The informed consent procedures needed for sample procurement should be clearly set, with the tiered or multi-layered model as the best choice. Donors should be allowed to decide whether they want to donate their samples for research purposes and if the sample would be used for all types of research, only for certain studies, or only for this specific research study. The previously collected material for diagnostic purposes could be used for research only with the approval of a research ethics committee and if it is irreversibly anonymized. The material previously collected for other biobanks could only be used for the research specified in previously collected informed consent procedures. In the future also the possibility of introduction of dynamic consent practices could be discussed. However, special attention should be paid to avoiding therapeutic misconceptions (9).

The work of the network should be coordinated with the Ministry of Health, Ministry of Science, Education and Sport, Ministry of War Veterans, and Ministry of the Interior since the biobanks in the consortium are related to their respective areas of work.

Our model of the Croatian National Centre for Biobanking is based on the model proposed for the Polish national DNA bank by Sak et al (6). Sak’s model is a model for a possible population biobank, but we have a different aim – brining all biobanking facilities together and improving the existing legal standards for biobanking in Croatia. We feel that our approach can be useful for small countries like Croatia. Why?

In the past Croatia was faced with similar challenges when it came to clinical trials. Before 2003, all clinical trials were reviewed locally by different research ethics committees in different hospitals. After 2003, new legal standards were created regulating this field and the review process of clinical trials was centralized at the Croatian Agency for Drugs and Medical Devices. This led to greater transparency of the review process, avoiding possible conflicts of interests. It created no waste of expertise and resources and allowed a better public control of the process (34). We, therefore, feel that the same benefit can be found in the networking of all biobanking facilities in Croatia.

Moreover, it seems that when it comes to biobanking practice in smaller countries like Slovenia or Cyprus there is a tendency to have a centralized national approach. This can bring together all the existing biobanking expertise and create better coordination and bigger research potential (35,36).

The Croatian National Centre for Biobanking with its proposed structure would be able to promote transparency and public interest in biobanking in Croatia, which in turn would enable biobanks to get more samples. It would safeguard ethical and quality standards in biobanking through harmonization and the ethical governance of the existing biobanking facilities. It would also prevent conflicts of interest that may arise in everyday work of biobanks. Finally, it would bring together all the existing resources to enhance research quality and facilitate exchange of samples and information under well regulated and transparent conditions within Croatia and with other countries.

In Europe new biobanks have been set up that will become part of international networks for exchange and research. National systems for collection and exchange of tissue samples and related material have also been developed around the world. It is not clear whether these national biobanking initiatives overlap or reproduce efforts which are being under way in other countries or even in their own country. When it comes to national biobanking schemes, the issues of sustainability and excessive financial costs are also being raised (16). We hope that with the Croatian National Centre for Biobanking we would be able to avoid these pitfalls.

We also hope that scientific community in Croatia will put forward their expert opinion about our proposal and that our ideas will lead to further discussions and actions on the part of all involved in the field of biobanking in Croatia as well as the Croatian government.

## 

**Table:** Figure 1. The Croatian National Center for Biobanking – organizational structure.
